# Differences in associations between family functioning and anxious and depressive symptoms in Chinese women and their partners in early pregnancy

**DOI:** 10.1192/bjo.2022.549

**Published:** 2022-08-19

**Authors:** Xuemei Qin, Shuyin Xu, Mohan Ma, Xing Fan, Xueqing Nie, Yan Zhang, Bangshan Liu, Lingjiang Li

**Affiliations:** Department of Psychiatry, National Clinical Research Center for Mental Disorders, The Second Xiangya Hospital of Central South University, Changsha 410011, Hunan, China; and Mental Health Institute of Central South University, China National Technology Institute on Mental Disorders, Hunan Technology Institute of Psychiatry, Hunan Key Laboratory of Psychiatry and Mental Health, Hunan Medical Center for Mental Health, Changsha 410011, Hunan, China; Changsha Hospital for Maternal and Child Health Care, Changsha 410011, Hunan, China

**Keywords:** Family functioning, anxious and depressive symptoms, women, partners, early pregnancy

## Abstract

**Background:**

Family functioning is associated with anxiety and depression. Perinatal depression and anxiety are common and influence the well-being of women, partners and their offspring. However, little is known about differences in associations between family functioning and mood symptoms in women and their partners in early pregnancy.

**Aims:**

Investigating differences in associations between family functioning and anxious and depressive symptoms in women and their partners in early pregnancy.

**Method:**

In total, 171 woman–partner pairs were enrolled. The Edinburgh Postnatal Depression Scale (EPDS), Patient Health Questionnaire-9 (PHQ-9), Generalized Anxiety Disorder 7-Item scale (GAD-7) and Family Assessment Device (FAD) were performed. Correlation analysis and multiple linear regression analysis were applied to investigate the associations.

**Results:**

In pregnant women, all the subscale scores on the FAD were correlated with total scores on the EPDS and GAD-7 (all *P* < 0.05), whereas only the Roles subscale showed a predicting effect in regression models (*P* < 0.01). In partners, all the subscale scores on the FAD were related to total scores on the PHQ-9 (all *P* < 0.05), whereas only the Problem Solving subscale showed a predicting effect (*P* = 0.029).

**Conclusions:**

Our findings indicate that family functioning in the domain of roles is associated with anxious and depressive symptoms in pregnant women and functioning in the domain of problem solving is associated with depressive symptoms in partners. Clinicians should pay special attention to roles and problem solving when assessing mood symptoms in pregnant women and their partners. This study also provides a basis for family health education in early pregnancy.

The family functions as a unit. Family functioning refers to whether the family can complete a series of tasks that provides conditions for the healthy development of family members.^1^ According to the McMaster Model of Family Functioning, it can be described in terms of seven domains: problem solving, communication, roles, affective responsiveness, affective involvement, behaviour control and general functioning.^2^ Impairment of family functioning is the inability of a family to adapt to changing circumstances and balance the needs of family members with the whole domestic system.^3^ Family functioning plays an important role in the mental health of family members. According to previous studies, poor family functioning is correlated with depression and anxiety. It influences clinical course,^4^ recovery^5^ and relapse of depression^6^ in adolescents,^7^ college students^8^ and older adults.^9^

Pregnancy is an important event in family life and is prone to exacerbating family dysfunction. Associations between poor family functioning and depression in women in the second and third trimester of pregnancy^10,11^ and perinatal anxious symptoms have been found.^12^ Generally, changes in the capacity of any family member to contribute may influence the whole family and its members.^13^ In early pregnancy, women suffer from obvious physical discomfort (such as nausea and vomiting)^14^ and psychological distress,^15^ which may disturb their contribution and further influence family functioning. Meanwhile, partners are the main caregivers of pregnant women and often play a role different from the women in a family. Therefore, associations between family functioning and anxious and depressive symptoms in pregnant women and partners may be distinct, which has been rarely discussed before and was the objective of the current study. We hypothesised that there were significant differences in associations between family functioning and anxious and depressive symptoms in Chinese pregnant women and their partners in early pregnancy.

## Method

### Participants

In total, 283 pregnant women at their initial prenatal visit to Changsha Hospital for Maternal and Child Health Care, Changsha, China, were screened by a psychiatrist between 29 December 2020 and 23 July 2021, and 197 partners of these pregnant women who agreed to participate in the current study were enrolled. The following inclusion criteria for all participants were: (a) Han ethnicity; (b) education years ≥9. The additional inclusion criteria for the pregnant women were: (a) aged between 18 and 40 years; (b) in the first trimester of pregnancy (gestational weeks less than 14); (c) living with a partner. Participants with current medical conditions (such as endocrine and metabolic diseases, neurological system diseases or brain injury) or any history of psychiatric disorder except anxiety and depression were excluded. Finally, 171 woman–partner pairs were enrolled in the data analyses.

The authors assert that all procedures contributing to this work comply with the ethical standards of the relevant national and institutional committees on human experimentation and with the Helsinki Declaration of 1975, as revised in 2008. All procedures involving human participants/patients were approved by the ethics committee of the Second Xiangya Hospital of Central South University (approval number: PND202008). All participants gave written informed consent to participate in this study.

### Assessment

A questionnaire designed for the study was used to collect general demographic information. To assess depressive and anxious symptoms, the Edinburgh Postnatal Depression Scale (EPDS), Patient Health Questionnaire-9 (PHQ-9) and Generalized Anxiety Disorder 7-Item scale (GAD-7) were administered to the pregnant women. The PHQ-9 and GAD-7 were administered to their partners. The Family Assessment Device (FAD) was administered to both the women and their partners to assess family functioning individually. The women completed these scales on site and their partners completed them online.

#### EPDS

The EPDS is the most widely used scale for the detection of perinatal depression. It is rated on a four-point Likert scale ranging from never (0) to very often (3). Participants choose the response most correlated with their feelings in the past week. The validity and reliability of the Chinese version of the EPDS were validated to be satisfactory.^16^

#### PHQ-9

The PHQ-9 has been demonstrated to be reliable and valid.^17^ Participants are asked how often they have been bothered by the nine problems over the past 2 weeks. The total score on this scale is used to determine depression severity.

#### GAD-7

The GAD-7 asks participants how often they have been bothered by the seven items over the past 2 weeks. This scale shows good reliability and validity.^18^

#### FAD

The FAD assesses family functioning and is based on the McMaster Model of Family Functioning.^19^ The reliability and validity of the Chinese version of the FAD are good.^20^

The FAD consists of 60 self-report items. Participants choose to answer ‘strongly agree’, ‘agree’, ‘disagree’ or ‘strongly disagree’ for each item. This scale can be divided into seven subscales: Problem Solving (the ability of the family to solve problems to maintain effective family functioning), Communication (how the family verbally exchanges information in instrumental and effective areas), Roles (the family's behavioural patterns to accomplish family functioning, such as the provision of resources and support, or development of life skills), Affective Responsiveness (whether a family can respond to affective stimuli with appropriate feelings), Affective Involvement (the degree to which family members are concerned about and value each other's activities), Behaviour Control (the way in which a family handles the behaviours of its members in various situations, such as social or dangerous situations, and expresses and meets members’ needs and drives), and General Functioning (common interaction of the whole family).^2,21^ Higher scores indicate worse family functioning.

To assess the common individual-level variance of perception of family functioning in a family,^22^ we used mean subscale scores on the FAD obtained by combining the subscale scores of the pregnant women with those of their partners in the data analyses. The use of mean FAD was consistent with a prior study.^23^

### Data analysis

Data are presented as mean (s.d.). Data analyses were done using IBM SPSS Statistics version 20 for Windows. Paired-sample *t*-tests and McNemar tests were used to compare general demographic and clinical information between the pregnant women and their partners. Correlation analyses were performed to investigate correlations of demographic variables and mean subscale scores on the FAD with anxious and depressive symptoms in participants. Variables simultaneously significant in correlation analyses and satisfying the conditions of the regression analysis were entered in the multiple linear regression analyses of mood symptoms. Statistical significance was defined as a two-tailed *P* < 0.05.

## Results

### General demographic information and clinical characteristics

Sociodemographic and clinical information are shown in Table 1. The mean age of the pregnant women was about 29 years. All of them were in the first trimester of pregnancy and their mean duration of pregnancy was about 48 days; 61.4% had a planned pregnancy and 57.3% had experienced early pregnancy reactions. A small proportion had a history of depression (2.3%). The majority of the women and their partners were married (85.4%) and employed (>71.0%). The mean years in education for the women and their partners were balanced (*t* = −1.379, *P* = 0.170). Nearly a half of the partners had a history of drinking (53.8%) and smoking (48.0%). The proportions of partners with a history of drinking and smoking were significantly higher than those for the pregnant women (both *P* < 0.001).

**Table 1:** General demographic information and clinical characteristics in pregnant women and their partners

Item	Pregnant women (*n* = 171)	Partners (*n* = 171)	*t* or *χ^2^*	*P*^a^
Age, years: mean (s.d.)	29.20 (4.17)	31.39 (4.56)	−9.040	**<0.001**^b^
BMI, kg/m^2^: mean (s.d.)	21.58 (3.19)	23.81 (3.37)	−7.406	**<0.001**^b^
Education, years: mean (s.d.)	14.96 (2.33)	15.19 (2.51)	−1.379	0.170^b^
Married, *n* (%)	146 (85.4%)	146 (85.4%)	–	–
Employed, *n* (%)	122 (71.3%)	169 (98.8%)	–	**<0.001**^c^
Monthly income ≥5000 RMB, *n* (%)	99 (57.9%)	165 (96.5%)	–	**<0.001**^c^
History of drinking, *n* (%)	9 (5.3%)	92 (53.8%)	–	**<0.001**^c^
History of smoking, *n* (%)	6 (3.5%)	82 (48.0%)	–	**<0.001**^c^
Duration of pregnancy, days: mean (s.d.)	48.20 (11.99)	–	–	–
Planned pregnancy, *n* (%)	105 (61.4%)	–	–	–
Early pregnancy reactions, *n* (%)	98 (57.3%)	–	–	–
History of previous live birth(s), *n* (%)	45 (26.3%)	–	–	–
History of depression, *n* (%)	4 (2.3%)	–	–	–
History of anxiety, *n* (%)	1 (0.6%)	–	–	–
PHQ-9 total score, mean (s.d.)	5.23 (4.07)	4.94 (4.90)	0.624	0.534^b^
GAD-7 total score, mean (s.d.)	3.82 (3.34)	3.86 (4.26)	−0.093	0.926^b^
EPDS total score, mean (s.d.)	6.96 (3.98)	–	–	–

BMI, body mass index; PHQ-9, Patient Health Questionnaire-9; GAD-7, Generalized Anxiety Disorder 7-Item scale; EPDS, Edinburgh Postnatal Depression Scale.

a. Bold values indicate statistical significance.

b. Paired-sample *t*-test.

c. McNemar test.

The mean score on the PHQ-9 was approximately 5 points for both the pregnant women and their partners. The mean score on the GAD-7 was approximately 3.8 points for both groups. The mean total scores on the EPDS in pregnant women was about 7 points. There was no statistical difference in mean total scores on the PHQ-9 and GAD-7 between the two groups (both *P* > 0.05, Table 1). Mean subscale scores on the FAD are shown in Table 2.

**Table 2:** Mean subscale scores on the Family Assessment Device (FAD) in woman–partner pairs (*n* = 171)

Item	Mean	s.d.
FAD-PS	1.92	0.31
FAD-CM	2.04	0.35
FAD-RL	2.10	0.30
FAD-AR	2.02	0.36
FAD-AI	2.06	0.35
FAD-BC	2.20	0.22
FAD-GF	1.80	0.35

PS, Problem Solving; CM, Communication; RL, Roles; AR, Affective Responsiveness; AI, Affective Involvement; BC, Behaviour Control; GF, General Functioning.

### Correlations of demographic variables and mean subscale scores on the FAD with mood symptoms in pregnant women and their partners

Variables related to total scores on the EPDS, PHQ-9 and GAD-7 for all participants are shown in Table 3. In the pregnant women, age had a negative correlation with total scores on the EPDS (*r* = −0.229, *P* < 0.01) and history of depression was positively correlated with total scores on the GAD-7 (*r* = 0.164, *P* < 0.05). In partners, history of smoking and education were correlated with total scores on the PHQ-9 and GAD-7 respectively (*r* = 0.165 and −0.160 respectively; both *P* < 0.05).

**Table 3:** Correlations of demographic variables and mean subscale scores on the Family Assessment Device (FAD) with mood symptoms in pregnant women and their partners

Item	Pregnant women (*n* = 171)		Partners (*n* = 171)	
	EPDS	GAD-7	PHQ-9	GAD-7
Age^a^	−0.229**	−0.130	−0.048	−0.034
BMI^a^	−0.035	−0.037	−0.140	−0.088
Education^a^	−0.146	−0.102	−0.081	**−0**.160*
Marital status^b^	−0.072	−0.068	−0.149	−0.110
Employed^b^	0.007	0.001	−0.035	0.006
Monthly income^b^	−0.052	−0.021	0.006	0.006
History of drinking^b^	0.057	−0.028	0.083	0.093
History of smoking^b^	0.007	0.021	**0**.165*	0.083
Duration of pregnancy^a^	−0.045	−0.038	–	–
Planned pregnancy^b^	−0.139	−0.076	–	–
Early pregnancy reactions^b^	0.098	0.066	–	–
History of previous live birth(s)^b^	0.005	0.002	–	–
History of depression^b^	0.101	**0**.164*	–	–
History of anxiety^b^	0.130	0.132	–	–
FAD-PS^c^	**0**.286***	**0**.360***	**0**.353***	**0**.246**
FAD-CM^c^	**0**.366***	**0**.389***	**0**.353***	**0**.296***
FAD-RL^c^	**0**.492***	**0**.486***	**0**.292***	**0**.262**
FAD-AR^c^	**0**.323***	**0**.335***	**0**.173*	0.138
FAD-AI^c^	**0**.352***	**0**.325***	**0**.228**	**0**.268***
FAD-BC^c^	**0**.192*	**0**.186*	**0**.260**	**0**.267***
FAD-GF^c^	**0**.440***	**0**.454***	**0**.311***	**0**.310***

EPDS, Edinburgh Postnatal Depression Scale; GAD-7, Generalized Anxiety Disorder 7-Item scale; PHQ-9, Patient Health Questionnaire-9; BMI, body mass index; PS, Problem Solving; CM, Communication; RL, Roles; AR, Affective Responsiveness; AI, Affective Involvement; BC, Behaviour Control; GF, General Functioning.

a. Pearson correlation analysis

b. Spearman correlation analysis.

c. Pearson partial correlation analysis.

Data are presented as *r* coefficients; bold values indicate statistical significance: *P_r_ < 0.05, **P_r_ < 0.01, ***P_r_ < 0.001.

All the mean subscale scores on the FAD were correlated with total scores on the EPDS and GAD-7 in pregnant women and with total scores on the PHQ-9 in partners (all *P* < 0.05). FAD subscales Problem Solving, Communication, Roles, Affective Involvement, Behaviour Control and General Functioning were associated with total scores on the GAD-7 in partners (all *P* < 0.05). There was no significant relationship between FAD Affective Responsiveness and total scores on the GAD-7 in partners (*r* = 0.138, *P* > 0.05).

### Multiple linear regression analyses of mood symptoms in pregnant women

Results of regression analyses of total scores on the EPDS and GAD-7 in the pregnant women are shown in Table 4. In the regression model for EPDS scores, only age (standardised regression coefficient *β* = −0.181; *P* = 0.006) and the FAD Roles subscale scores (*β* = 0.385, *P* < 0.001) had statistical significance. This model could explain 33.2% of the variation in total scores on the EPDS (adjusted *R*^2^ = 0.332, *P* < 0.001). History of depression (*β* = 0.224, *P* = 0.001) and FAD Roles (*β* = 0.341, *P* = 0.002) had a significant prediction effect for GAD-7 scores and this model could explain 30.7% of the variation in GAD-7 total scores (adjusted R^2^ = 0.307, *P* < 0.001).

**Table 4:** Multiple linear regression analyses of mood symptoms in pregnant women (*n* = 171).

Dependent variable	Independent variable	*β*	*t*	*P* for *β*	Adjusted *R*^2^	*F*	*P*
EPDS	Age	−0.181	−2.781	**0**.**006**	0.332	11.358	**<0**.**001**
	FAD-PS	0.052	0.581	0.562			
	FAD-CM	0.002	0.018	0.986			
	FAD-RL	0.385	3.718	**<0**.**001**			
	FAD-AR	0.006	0.060	0.952			
	FAD-AI	−0.028	−0.274	0.785			
	FAD-BC	−0.015	−0.213	0.831			
	FAD-GF	0.206	1.307	0.193			
GAD-7	History of depression	0.224	3.444	**0**.**001**	0.307	10.283	**<0**.**001**
	FAD-PS	0.151	1.669	0.097			
	FAD-CM	−0.064	−0.482	0.630			
	FAD-RL	0.341	3.215	**0**.**002**			
	FAD-AR	0.001	0.008	0.993			
	FAD-AI	−0.041	−0.392	0.696			
	FAD-BC	−0.046	−0.631	0.529			
	FAD-GF	0.244	1.520	0.130			

EPDS, Edinburgh Postnatal Depression Scale; GAD-7, Generalized Anxiety Disorder 7-Item scale; FAD, Family Assessment Device; PS, Problem Solving; CM, Communication; RL, Roles; AR, Affective Responsiveness; AI, Affective Involvement; BC, Behaviour Control; GF, General Functioning.

Bold values indicate statistical significance.

### Multiple linear regression analyses of mood symptoms in partners

Results of linear regression analyses of total scores on the PHQ-9 and GAD-7 in the partners are shown in Table 5. In the regression model for PHQ-9 scores, only FAD Problem Solving was significant (*β* = 0.217, *P* = 0.029). This model could explain 18.1% of the variation in total scores on the PHQ-9 (adjusted *R*^2^ = 0.181, *P* < 0.001). In the model for GAD-7 scores, no variables showed statistical significance (all *P* > 0.05).

**Table 5:** Multiple linear regression analyses of mood symptoms in partners (*n* = 171)

Dependent variable	Independent variable	*β*	*t*	*P* for *β*	Adjusted *R*^2^	*F*	*P*
PHQ-9	History of smoking	0.121	1.628	0.105	0.181	5.680	**<0**.**001**
	FAD-PS	0.217	2.205	**0**.**029**			
	FAD-CM	0.277	1.905	0.059			
	FAD-RL	0.121	1.043	0.298			
	FAD-AR	−0.233	−1.962	0.052			
	FAD-AI	−0.026	−0.223	0.824			
	FAD-BC	0.115	1.404	0.162			
	FAD-GF	−0.001	−0.004	0.997			
GAD-7	Education	−0.078	−1.014	0.312	0.112	4.071	**<0**.**001**
	FAD-PS	0.083	0.808	0.420			
	FAD-CM	0.056	0.378	0.706			
	FAD-RL	0.012	0.101	0.920			
	FAD-AI	0.066	0.553	0.581			
	FAD-BC	0.152	1.823	0.070			
	FAD-GF	0.095	1.573	0.568			

PHQ-9, Patient Health Questionnaire-9; GAD-7, Generalized Anxiety Disorder 7-Item scale; FAD, Family Assessment Device; PS, Problem Solving; CM, Communication; RL, Roles; AR, Affective Responsiveness; AI, Affective Involvement; BC, Behaviour Control; GF, General Functioning.

Bold values indicate statistical significance.

### Correlations of items of the FAD Roles subscale with mood symptoms in pregnant women

Associations between items of the FAD Roles subscale and anxious and depressive symptoms in the pregnant women are shown in Table 6. The items ‘We make sure members meet their family responsibilities’, ‘Family tasks don't get spread around enough’, ‘We have trouble meeting our bills’, ‘Little time to explore personal interests’, ‘If people are asked to do something, they need reminding’, ‘We are generally dissatisfied with the family duties assigned to us’ and ‘Not having reasonable means of transport’ were all related to total scores on the EPDS and GAD-7 in the women (all *P* < 0.05). The item ‘Sometimes running out of things that we need’ was correlated with total scores only on the EPDS (*r* = 0.175, *P* < 0.05).

**Table 6:** Correlations of items of the Family Assessment Device Roles subscale with mood symptoms in pregnant women (*n* = 171)

Item	EPDS	GAD-7
4. When you ask someone to do something, you have to check that then did it^a^	0.085	0.131
8. Sometimes running out of things that we need^a^	0.175*	0.118
10. We make sure members meet their family responsibilities	**0**.342***	**0**.327***
15. Family tasks don't get spread around enough^a^	**0**.243**	**0**.270***
23. We have trouble meeting our bills^a^	**0**.320***	**0**.214**
30. Each of us has particular duties and responsibilities	0.122	0.066
34. Little time to explore personal interests^a^	**0**.301***	**0**.252**
40. We discuss who is to do household jobs	0.047	−0.015
45. If people are asked to do something, they need reminding^a^	**0**.251**	**0**.220**
53. We are generally dissatisfied with the family duties assigned to us^a^	**0**.286***	**0**.289***
58. Not having reasonable means of transport^a^	**0**.252**	**0**.221**

EPDS, Edinburgh Postnatal Depression Scale; GAD-7, Generalized Anxiety Disorder 7-Item scale.

a. Items need reverse scoring.

Data are presented as *r* coefficients; bold values indicate statistical significance: *P_r_ < 0.05, **P_r_ < 0.01, ***P_r_ < 0.001.

### Correlations of items of the FAD Problem Solving subscale with depressive symptoms in partners

Correlations of items of the FAD Problem Solving subscale with total scores of PHQ-9 in partners are shown in Table 7. the items ‘We resolve most everyday problems around the house’, ‘We usually act on our decisions regarding problems’, ‘We resolve most emotional upsets that come up’, ‘We confront problems involving feelings’ and ‘We try to think of different ways to solve problems’ were correlated with total scores on the PHQ-9 in partners (all *P* < 0.01).

**Table 7:** Correlations of items of the Family Assessment Device Problem Solving subscale with depressive symptoms in partners (*n* = 171)

Item	PHQ-9
2. We resolve most everyday problems around the house	0.236**
12. We usually act on our decisions regarding problems	**0**.200**
24. After our family tries to solve a problem, we usually discuss whether it worked or not	0.034
38. We resolve most emotional upsets that come up	**0**.350***
50. We confront problems involving feelings	**0**.285***
60. We try to think of different ways to solve problems	**0**.246**

PHQ-9, Patient Health Questionnaire-9.

Data are presented as *r* coefficients; bold values indicate statistical significance; **P_r_ < 0.01, ***P_r_ < 0.001.

## Discussion

To our best knowledge, the current study is the first to investigate differences in associations between family functioning and anxious and depressive symptoms in Chinese women and their partners in early pregnancy. We observed that in the first trimester of pregnancy: (a) family functioning in the domain of roles made an independent contribution to depressive and anxious symptoms in pregnant women and (b) family functioning in the domain of problem solving made an independent contribution to depressive symptoms in partners. These findings highlight the need to pay special attention to roles and problem solving in the assessment of mood symptoms in Chinese pregnant women and their partners respectively.

### Associations between roles and anxious and depressive symptoms in women in early pregnancy

The roles domain of the McMaster Model of Family Functioning^2^ refers to patterns of behaviour to fulfil necessary family functions, including provision of resources, and is reflected on the FAD Roles subscale in items such as ‘We have trouble meeting our bills’ or ‘Not having reasonable means of transport’. According to previous studies, socioeconomic conditions are risk factors for depression and anxiety.^24^ People in communities with the lowest incomes have a higher frequency of depression and anxiety.^25^ The independent contribution of roles to mood symptoms and correlations of items of the Roles subscale related to providing resources with these symptoms in the current study support the results of the previous studies. It also pointed out that better family functioning in the provision of resources may be beneficial to the mental health of pregnant women in early pregnancy.

The Roles subscale of the FAD evaluates role allocation and role accountability.^19^ The better and clearer the role allocation and processes of accountability, the healthier the family.^2^ In the present study, items related to role allocation such as ‘We make sure members meet their family responsibilities’, ‘Family tasks don't get spread around enough’ and ‘We are generally dissatisfied with the family duties assigned to us’ were all significantly correlated with depressive and anxious symptoms in pregnant women.

Normally, women are reported to take more responsibility for household tasks than men.^26^ On the one hand, perceptions of equity and time spent on housework are related to depression in women.^27^ On the other hand, adherence to traditional female roles^28^ and the identity of a housewife are associated with prenatal anxiety.^29^ Results of our study showed that the assignment of family tasks may have failed to readjust in early pregnancy, and pregnant women, who usually suffer from physical discomfort,^14^ tend to hold a worse view of family functioning in the domain of roles, which seems to play an important role in their mood symptoms.

### Associations between problem solving and depressive symptoms in partners in early pregnancy

According to the McMaster Model, problem solving means the ability of a family to solve instrumental and affective problems effectively to maintain good family functioning.^2^ Family functioning in the domain of problem solving made a significant contribution to depressive symptoms in partners. Items of the FAD Problem Solving subscale such as ‘We resolve most emotional upsets that come up’, ‘We confront problems involving feelings’ and ‘We try to think of different ways to solve problems’ were all associated with depressive symptoms in partners in this study.

Generally, partners are the main caregivers of pregnant women but men tend to have limited knowledge about pregnancy.^30^ Results of our study revealed that solving instrumental and affective problems effectively to maintain family functioning in the domain of problem solving in early pregnancy may be challenging for them. Meanwhile, the only positive predictive effect of FAD Problem Solving scores on depressive symptoms in partners was different from the results of a previous study, which reported a significant predictive effect of general family functioning on depressive symptoms in caregivers of acute stroke survivors.^23^ The reason for the difference may lie in different populations, but they both support the effects of family functioning on depressive symptoms.

Consistent with the hypothesis of the current study, associations between family functioning and mood symptoms in pregnant women and their partners were different. However, the reasons for the differences are unknown. It is possibly because of their different social roles. According to social role theory, the characteristics and behaviours of men and women are different in daily life.^31^ Especially, common traits associated with the family role ascribed to women in China include being virtuous, which refers to being caring, gentle and good at doing housework and cooking.^32^ However, the execution of traditional roles such as household tasks for women may be influenced by physical and mental discomfort in early pregnancy. Meanwhile, the ‘maternal role competence’ of pregnant women has been reported to decline during early motherhood.^33^ Therefore, family roles may not be adjusted in a timely fashion in early pregnancy and this may cause psychological distress to pregnant women.

Maternal depression and anxiety are common during the perinatal period^34^ and lead to numerous negative consequences for mothers^35^ and their offspring.^36^ Furthermore, the perinatal period is positively associated with paternal depression and anxiety,^37,38^ which affects about 10% of men during that period.^37,39^ The results of the current study may be beneficial to guide targeted screening for anxious and depressive symptoms in pregnant women with poor family functioning in the domain of roles and for depressive symptoms in partners with poor family functioning in the domain of problem solving in the first trimester of pregnancy.

### Limitations

Some deficiencies of this study should be mentioned when interpreting these results. First, we only enrolled women (and their partners) in the first trimester of pregnancy. Whether the findings can be applied to the second or third trimesters or the postpartum period is unknown. Second, we assessed family functioning using the self-reported FAD. The clinician-rated McMaster Clinical Rating Scales and clinician-directed McMaster Structured Interview were not involved. Third, the reasons why associations between family functioning and mood symptoms were different in pregnant women and their partners are unknown, which needs to be investigated in the future.

## Data Availability

The data that support the findings of this study are available on reasonable request from the corresponding author.
